# Corneal densitometry: an innovative method to quantitatively evaluate corneal changes after phacovitrectomy

**DOI:** 10.1186/s12886-023-02818-3

**Published:** 2023-03-06

**Authors:** Xin Liu, Yuting Shao, Hui Lin, Chunyu Liu, Jiaqi Shen, Li Zhang, Yanlong Bi

**Affiliations:** 1 Department of Ophthalmology, Tongji Hospital, School of Medicine, Tongji University, No. 389, Xincun Road, Putuo District, Shanghai, 200000 China; 2 Department of Ophthalmology, Guizhou Provincial People’s Hospital, Guiyang, Guizhou China; 3 Tongji Eye Institute, School of Medicine, Tongji University, Shanghai, China

**Keywords:** Corneal densitometry, Endothelial cell density, Hexagonality, Coefficient of variation, Phacovitrectomy

## Abstract

**Background:**

To quantitatively investigate corneal changes and the correlation between corneal densitometry (CD) and endothelial parameters after phacovitrectomy.

**Methods:**

Thirty-eight eyes with idiopathic full-thickness macular holes (iFTMHs) and cataracts underwent phacovitrectomy. Examinations were conducted at baseline and Day 1, Day 7, Month 1, and Month 3 postoperatively. CD and central corneal thickness (CCT) were measured using Pentacam. Corneal endothelial cell density (ECD), coefficient of variation (CV), and hexagonality (HEX) were measured using specular microscopy.

**Results:**

ECD and HEX significantly decreased after surgery and the change in HEX occurred prior to CV. CCT increased immediately after surgery and recovered 3 months postoperatively. CD values increased significantly 1 day after surgery and then gradually decreased. For CD in the 0–2 mm zone, it took 1 month to recover in the central and posterior layers and 3 months in the anterior and total layers. For CD in the 2–6 mm zone, the central layer recovered at Day 7, the anterior and total layers recovered at 1 month, and the posterior layer did not recover until 3 months postoperatively. The CD within all layers in the 0–2 mm zone was positively correlated with CCT. Posterior CD in the 0–2 mm zone was negatively correlated with ECD and HEX.

**Conclusions:**

CD is not only correlated with CCT, ECD, and HEX but also reflects the state of the whole cornea and each layer. CD can be an objective, rapid, and noninvasive tool that reflects corneal health and undetectable edema and monitors the process of lesion repair.

**Trial registration:**

This study was registered with the Chinese Clinical Trial Registry (31/10/2021, ChiCTR2100052554).

## Background

Johnson and Gass first described the idiopathic full-thickness macular hole (iFTMH) in 1988 [1]. Vitrectomy was an effective management for iFTMH. However, more than 50% of patients develop obvious cataracts within 2 years after vitrectomy and require a second operation [2–4]. Previous studies have stated that phacovitrectomy (PPV) has good visual outcomes and anatomic closure rates in phakic patients with iFTMH [5, 6].

Corneal endothelial cells are essential for maintaining corneal transparency and visual performance. Endothelial cell loss (ECL) after cataract surgery has been widely reported [7–9]. However, ECL after combined phacovitrectomy without silicone or gas tamponade has not been widely reported. Corneal decompensation is a nonnegligible issue that impairs visual acuity after otherwise successful surgery, especially in patients with low preoperative endothelial cell density (ECD). In the real world, routine preoperative corneal endothelium microscopy only measures a small area of the central corneal endothelium which cannot reflect the entire corneal endothelium to some extent.

Corneal densitometry (CD) is performed by the built-in software of a high-resolution rotation Scheimpflug camera (Pentacam AXL, Oculus, Germany), which can noninvasively obtain the whole cornea data in seconds. CD is expressed in grayscale units (GSUs) from 0 (maximum transparency) to 100 (completely opaque) based on different corneal light backscattering. Studies have shown that the CD values can reflect corneal transparency [10, 11], undetectable edema [12], and endothelial health [13, 14].

The present study aimed to prospectively observe the whole cornea status using CD values, and to investigate the correlation between CD of the posterior layer and specular microscopic parameters after phacovitrectomy. To our knowledge, this is the first prospective study to evaluate corneal changes in different layers and different annuli after phacovitrectomy using CD values and to explore the correlation between CD values and specular microscopic parameters.

## Materials and methods

### Participants

This was a prospective single-arm study. Thirty-eight eyes of 36 patients with iFTMH combined cataract (Grade 2 or above according to the Lens Opacities Classification System III (LOCS III)) [15] who underwent phacovitrectomy surgery at the Department of Ophthalmology, Tongji Hospital affiliated with Tongji University School of Medicine between October 2021 and September 2022 were enrolled. Exclusion criteria were any ocular diseases other than iFTMH or cataract, contact lens wear, previous ocular surgery, diabetes, and connective diseases. The study was registered with the Chinese Clinical Trial Registry (31/10/2021, ChiCTR2100052554). Moreover, it was approved by the Ethics Committee of the Shanghai Tongji Hospital and followed the principles of the Declaration of Helsinki. All the participants provided written informed consent.

Ocular examinations, including best-corrected visual acuity (BCVA), intraocular pressure (IOP), slit-lamp examination, dilated indirect funduscopic examination and optical coherence tomography (OCT) (Carl Zeiss Meditec, Germany), were performed within 1 week before surgery and 1 day, 7 days, 1 month, and 3 months after surgery.

Corneal endothelial cells were examined using noncontact specular microscopy (SP-1P; Topcon, Japan). This device takes different azimuth shots of a 0.25 mm*0.55 mm corneal endothelial surface. The built-in software can then automatically provide various parameters. The variables included in our study were ECD, hexagonality (HEX), and coefficient of variation (CV).

CD and central corneal thickness (CCT) were provided by the appendix in Pentacam. The study was conducted in a windowless dark room. The ambient light level measured by an illuminometer (LT40; Extech Instruments Corp., Waltham, MA, USA) was approximately 5 lx. Density measurements were represented by standardized gray level units from 0 to 100. Zero represents maximum transparency and 100 represents minimum transparency. The software divided the cornea into four concentric radial areas: 0–2 mm, 2–6 mm, 6–10 mm, and 10–12 mm. Meanwhile, the cornea is divided into three layers, the anterior layer (superficial 120 µm), central layer (between anterior and posterior layers), and posterior layer (depth 60 µm) [16].

### Surgical technique

A single surgeon (Yanlong Bi) performed all the surgeries under retrobulbar anesthesia. Before the surgery, the conjunctiva and cornea within the operative field were disinfected with 5% povidone-iodine (Shanghai Likang Disinfection High-tech Co., Ltd., China) for 3 min. Cataract surgery was performed via a clear corneal incision, viscoelastic substance (IVIZ, Bausch & Lomb, USA) injection, anterior capsulorhexis, and phacoemulsification, followed by implantation of an intraocular lens (IOL) (ASPIRA-aAY, HumanOptics, Erlangen, Germany). After cataract surgery, standard and complete PPV was performed using a 25-gauge instrument. Next, the internal limiting membrane (ILM) was peeled 360° around the macular hole (MH) after staining with indocyanine green and then inserted into the MH smoothly. Sterile air was injected at the end of the vitrectomy. The patients were asked to maintain face-down positioning for at least 7 days after surgery.

### Follow-up examinations and outcome measures

Ocular examinations were conducted at 1, 7, 30, and 90 days postoperatively using the same assessments as at baseline. Pentacam and specular microscopic examinations were performed by the same technician at each visit. For each inspection, we calculated the average value of three consecutive measurements.

### Statistical analysis

Statistical analyses were performed using SPSS (version 20.0; IBM Corp., USA). Normal distribution was verified using the Shapiro‒Wilk test and Kolmogorov‒Smirnov test. Descriptive data are presented as the mean ± standard deviation or median [interquartile range]. Nonnormally distributed continuous variables were compared using the related samples of Friedman’s two-way analysis of variance by rank and adjusted with Bonferroni correction. We used Spearman’s correlation coefficient to analyze the correlation between CD and specular microscopic variables. Statistical significance was set at *p* < 0.05.

## Results

Thirty-eight eyes of 36 patients (17 men and 19 women) were enrolled in the study. The average age was 61.1 ± 7.6 years. All surgeries were completed successfully, and ocular hypertension (IOPs of 30, 28, and 25 mmHg) was found in three eyes and controlled with IOP-lowering medication. No other perioperative complications, such as dropped nucleus, anterior and posterior capsular rupture, vitreous hemorrhage, retinal detachment, or endophthalmitis were observed.

Table 1 summarizes the baseline and postoperative values of clinical biometric variables. BCVA (Log MAR) was improved in all patients after surgery. There was no significant difference in the IOP at each follow-up point. The CCT significantly increased on Day 1 after surgery and then gradually decreased until 3 months after surgery. The ECD significantly decreased immediately after surgery and progressively decreased over time. The average rates of ECL (ECL%) were 4.76 ± 4.64, 5.79 ± 6.12, 8.48 ± 7.15, and 11.49 ± 7.51 on Day 1, Day 7, Month1, and Month 3 after surgery, respectively. HEX significantly decreased at Day 1, Month 1 and Month 3 postoperatively. There was no significant difference in the CV at each follow-up point.

**Table 1:** Preoperative and postoperative data of patients enrolled in the study

Variables	Preoperative	Postoperative			
		Day 1	Day 7	Month 1	Month 3
BCVA (logMAR)	0.9 [0.7,1.0]	0.7 [0.5,1.00]	0.5 [0.4,0.8]	0.4 [0.3,0.7]	0.4 [0.3,0.7]
*p* value		0.817	< 0.001	< 0.001	< 0.001
IOP (mmHg)	14.0 [11.0,19.0]	17.5 [13.0,20.3]	15.0 [13.8,18.0]	13.5 [11.0,18.0]	14.5 [12.0,19.0]
*p* value		0.302	0.302	0.302	0.302
CCT (μm)	550.0 [541.3,557.0]	583.0 [576.5,597]	563.0 [550.8,570.3]	556.0 [544.0,561.3]	550.5 [540.0,560.0]
*p* value		< 0.001	< 0.001	0.004	0.952
ECD (cells/mm^2^)	2797.3 ± 389.7	2667.7 ± 216.5	2638.4 ± 240.2	2561.4 ± 240.4	2478.5 ± 257.1
*p* value		0.001	< 0.001	< 0.001	< 0.001
CV (%)	33.0 ± 3.7	33.8 ± 3.3	34.1 ± 2.8	33.6 ± 2.7	32.3 ± 2.8
*p* value		1	0.151	0.245	1
HEX (%)	56.0 [51.0,58.0]	54.5 [50.57]	54.5 [49.8,56]	53.0 [48.8,56.3]	53.0 [47.5,56.0]
*p* value		0.016	0.167	< 0.001	< 0.001

Data are presented as the mean ± standard deviation or median [interquartile range]

(*BCVA* Best-corrected visual acuity, *IOP* Intraocular pressure, *CCT* Central corneal thickness, *ECD* Endothelial cell density, *CV* Coefficient of variation in cell size, *HEX* percentage of hexagonal cells)

**Fig. 1:**
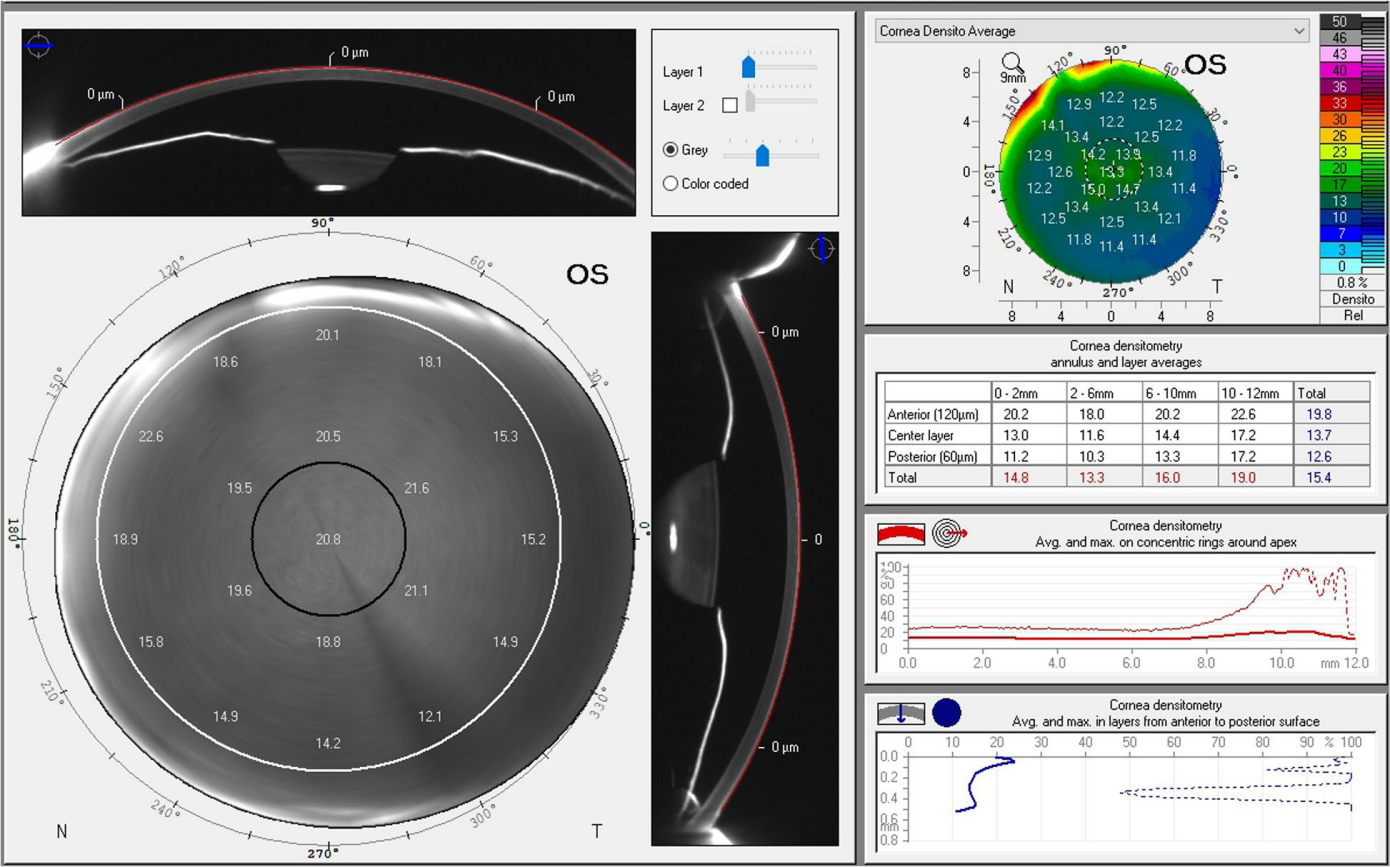
Shows the corneal densitometric examination of a patient 3 months after phacovitrectomy

The CD values are listed in Table 2. The CD values reached the highest point on Day 1 after surgery and gradually decreased over time. CD values in the 0–2 mm zone within the anterior layer were 20.31 ± 1.19 before surgery, and increased significantly on Day 1 (*p* < 0.001), on Day 7 (*p* < 0.001), and in Month 1 (*p* < 0.001) but recovered to normal in Month 3 (*p* = 0.952) after surgery. Meanwhile, CD values in the 2–6 mm and 0–6 mm zones within the anterior layer were significantly increased on Day 1 (*p* < 0.001) and on Day 7 (*p* < 0.001), but recovered to normal in Month 1 (*p* = 1.000) postoperatively. In the central layer, densitometry values increased significantly on the first day postoperatively but recovered to normal in Month 1 within 0–2 mm, on Day 7 within 2–6 mm, and in Month 1 within 0–6 mm. In the posterior layer, densitometric values obviously increased on Day 1 after surgery but recovered to normal in Month 1 within the 0–2 mm zone and in Month 3 within the 0–6 mm zone. However, the values did not recover until Month 3 in the 2–6 mm zone within the posterior layer. For total densitometry, the values were significantly increased on Day 1 after surgery but recovered in Month 1 in the 2–6 mm and 0–6 mm zones and in Month 3 in the 0–2 mm zone. Figure 1 shows the CD examination of a patient 3 months after phacovitrectomy.

**Table 2:** Preoperative and postoperative corneal densitometric values in patients enrolled in the study

Layer	Annulus (mm)	Preoperative	Postoperative			
			Day 1	Day 7	Month 1	Month 3
Anterior	0–2	20.4 [19.6,21.2]	25.4 [24.1,26.1]	23.0 [22.2,23.3]	21.9 [21.4,22.2]	20.9 [20.4,21.6]
	*p* value		< 0.001	< 0.001	< 0.001	0.952
	2–6	18.6 [18.1,20.0]	23.5 [22.5,24.5]	21.3 [20.1,21.8]	19.0 [18.5,20.2]	19.4 [18.6,20.2]
	*p* value		< 0.001	< 0.001	1.000	0.323
	0–6	19.7 [19.1,20.3]	24.4 [23.3,25.3]	22.1 [21.1,22.6]	20.4 [20.0,21.2]	20.2 [19.6,20.9]
	*p* value		< 0.001	< 0.001	0.545	0.545
Central	0–2	12.6 [11.9,13.1]	13.5 [13.2,14.1]	13.3 [13.0,13.4]	13.0 [12.7,13.1]	13.0 [12.6,13.2]
	*p* value		< 0.001	< 0.001	1.000	0.052
	2–6	11.8 [11.3,12.3]	12.7 [12.3,13.4]	12.3 [11.9,12.4]	12.1 [11.9,12.6]	12.1 [11.7,12.5]
	*p* value		< 0.001	1.000	1.000	1.000
	0–6	12.3 [11.9,12.6]	13.1 [12.8,13.7]	12.8 [12.4,12.9]	12.1 [11.9,12.6]	12.6 [12.1,12.8]
	*p* value		< 0.001	0.008	1.000	0.387
Posterior	0–2	10.0 [9.3,10.4]	10.9 [10.4,11.2]	10.7 [10.5,11.0]	10.4 [10.1,10.6]	10.2 [10.0,10.4]
	*p* value		< 0.001	< 0.001	0.052	1
	2–6	9.6 [9.2,10.1]	10.0 [9.6,10.5]	10.2 [9.8,10.4]	10.2 [9.8,10.4]	10.0 [9.8,10.5]
	*p* value		0.023	< 0.001	< 0.001	0.037
	0–6	9.9 [9.4,10.2]	10.5 [10.0,10.7]	10.5 [10.2,10.7]	10.3 [10.0,10.5]	10.2 [10.0,10.3]
	*p* value		< 0.001	< 0.001	0.016	0.111
Total	0–2	14.3 [13.7,14.8]	16.5 [16.1,17.5]	15.6 [15.2,15.8]	15.1 [14.8,15.3]	14.7 [14.4,15.0]
	*p* value		< 0.001	< 0.001	0.01	0.697
	2–6	13.4 [12.9,14.1]	15.5 [15.0,16.2]	14.6 [13.9,14.8]	13.8 [13.4,14.3]	13.9 [13.4,14.3]
	*p* value		< 0.001	< 0.001	1.000	0.545
	0–6	13.9 [13.5,14.3]	16.1 [15.6,16.8]	15.1 [14.6,15.3]	14.4 [14.1,14.7]	14.3 [13.9,14.6]
	*p* value		< 0.001	< 0.001	1.000	0.882

Data are presented as the mean ± standard deviation or median [interquartile range]

Table 3 shows the correlation analysis between the CD values and specular microscopic variables. CD values in the 0–2 mm and 2–6 mm zones within the posterior layer were negatively correlated with ECD (r_s_ = -0.241, *p* = 0.01; r_s_ = -0.177, *p* = 0.015). There was no significant correlation between the CD and CV. Densitometry values in the central 2 mm zone within the posterior layer correlated negatively with HEX ( r_s_ = -0.157, *p* = 0.031). CCT was positively correlated with all layers in the zone 0–2 mm, 2–6 mm, or 0–6 mm, except in the 2–6 mm zones within the posterior layer.

**Table 3:** Correlation between the specular microscopic parameters and corneal densitometric values in patients enrolled in the study

	ECD (cells/mm^2^)	CV (%)	HEX (%)	CCT(μm)
Anterior (0–2 mm)	r_s_ = 0.044	r_s_ = 0.029	r_s_ = 0.084	r_s_ = 0.645
	*p* = 0.550	*p* = 0.693	*p* = 0.251	*p* < 0.01
Central (0–2 mm)	r_s_ = 0.032	r_s_ = 0.017	r_s_ = 0.131	r_s_ = 0.487
	*p* = 0.658	*p* = 0.821	*p* = 0.071	*p* < 0.01
Posterior (0–2 mm)	r_s_ = -0.241	r_s_ = -0.045	r_s_ = -0.157	r_s_ = -0.213
	*p* = 0.01	*p* = 0.535	*p* = 0.031	*p* = 0.03
Total (0–2 mm)	r_s_ = 0.062	r_s_ = 0.038	r_s_ = 0.076	r_s_ = 0.590
	*p* = 0.392	*p* = 0.603	*p* = 0.297	*p* < 0.01
Anterior (2–6 mm)	r_s_ = 0.017	r_s_ = -0.014	r_s_ = 0.092	r_s_ = 0.644
	*p* = 0.820	*p* = 0.843	*p* = 0.205	*p* < 0.01
Central (2–6 mm)	r_s_ = -0.064	r_s_ = 0.019	r_s_ = 0.105	r_s_ = 0.530
	*p* = 0.378	*p* = 0.791	*p* = 0.148	*p* < 0.01
Posterior (2–6 mm)	r_s_ = -0.177	r_s_ = 0.007	r_s_ = 0.060	r_s_ = 0.125
	*p* = 0.015	*p* = 0.927	*p* = 0.409	*p* = 0.086
Total (2–6 mm)	r_s_ = -0.019	r_s_ = 0.006	r_s_ = 0.101	r_s_ = 0.615
	*p* = 0.791	*p* = 0.930	*p* = 0.166	*p* < 0.01
Anterior (0–6 mm)	r_s_ = 0.035	r_s_ = 0.001	r_s_ = 0.093	r_s_ = 0.660
	*p* = 0.631	*p* = 0.993	*p* = 0.200	*p* < 0.01
Central (0–6 mm)	r_s_ = -0.015	r_s_ = 0.028	r_s_ = 0.143	r_s_ = 0.546
	*p* = 0.834	*p* = 0.706	*p* = 0.049	*p* < 0.01
Posterior (0–6 mm)	r_s_ = -0.046	r_s_ = 0.069	r_s_ = -0.003	r_s_ = 0.201
	*p* = 0.529	*p* = 0.344	*p* = 0.964	*p* = 0.005
Total (0–6 mm)	r_s_ = 0.019	r_s_ = 0.014	r_s_ = 0.105	r_s_ = 0.634
	*p* = 0.798	*p* = 0.845	*p* = 0.151	*p* < 0.01

*ECD* Endothelial cell density, *CV* Coefficient of variation in cell size, *HEX* Percentage of hexagonal cells, *CCT* Central corneal thickness

## Discussion

In this prospective study, we investigated CD changes after phacovitrectomy surgery. To the best of our knowledge, this is the first study to measure the changes in CD after phacovitrectomy and to explore the correlation between CD and specular microscopic variables.

Our perioperative data showed that the anterior layer had the highest densitometric values, followed by the stroma and the lowest endothelium. Similar tendencies were proven in the literature using a Pentacam, [12, 13, 17, 18] confocal microscope [19, 20], and scatterometer, [21] making our measured values more reliable.

As the specular microscopic data we used were collected from the corneal apex, we focused on the posterior CD value in the zone 0–2 mm. The change in total CD in the central 2 mm had the same tendency as that of CCT, an indicator of edema, for transient increase on the first day after surgery and then decreased to normal in Month 1. This is consistent with the study of Hamoudi et al. [22]. According to depth, CD values in the anterior, central, and posterior layers represent the epithelial, stromal, and endothelial layers of the cornea, respectively. From our data, densitometric values in the central and posterior layers in the 0–2 mm zone decreased to normal on Day 7 after surgery, while the CCT and anterior layer in the 0–2 mm zone took 1 month even though slit-lamp examination showed no corneal epithelial edema 1 week after surgery. These above data indicated that the epithelial edema subsided slower than the stroma and the endothelium, suggesting that CD could detect subclinical edema, which slit lamp could not. This may be one of the reasons for postoperative visual acuity fluctuations. Ishikawa et al. enrolled 34 (54 eyes) Asian patients aged 71 ± 8.4 years old with age-related cataracts who underwent phacoemulsification and IOL implantation. They also found that densitometry in the anterior layer increased the most and recovered the last after cataract surgery. However, anterior densitometry in the 0–2 mm zone decreased to normal on Day 7 after surgery in their study, while it decreased to normal in Month 1 in our study [12]. The difference might account for the longer operation time and higher povidone iodine concentrations (5% vs. 1%) in our study [23]. The above findings reminded us that 1% povidone iodine brought more benefit to the epithelium, especially in patients with preoperative epithelial lesions.

ECD loss after phacoemulsification is an indisputable fact [7–9]. However, it is still controversial whether phacovitrectomy without silicone or gas as a tamponade aggravates corneal endothelial cell injury. To improve accuracy, we used only images that counted more than 75 cells, which were considered qualified, as Doughty et al. did [24]. In our study, ECL% reached 11.49 ± 7.51% at 3 months postoperatively (*p* < 0.01). A prospective Canadian study reported that phacovitrectomy caused slightly higher ECL% than PPV only (13.9% ± 15.5% vs. 9.0% ± 14.6%, *p* = 0.10), while combined surgery provided a greater visual benefit in patients with cataracts (*p* = 0.04) [25]. HEX, an indicator of pleomorphism, significantly decreased from the first month postoperatively. However, the CV, an indicator of polymegathism, hardly changed. Previous research also found that HEX statistically differed from baseline at 1 and 3 months postoperatively compared to CV after phacovitrectomy [22]. Cho et al. also reported the same results [17]. These results suggested that, compared with CV, HEX was more likely to be sensitive in reflecting ECL.

Another important issue in our study was the correlation between the CD in the posterior layer and specular microscopic values. In our study, we focused on assessing densitometry in the central 0–2 mm zone, as we examined the corneal endothelium in the central 0.25 mm*0.55 mm range. Recently, CD has been regarded as an objective and noninvasive tool to monitor corneal endothelial health [16, 18]. Tekin and Karmiris found a correlation between CD and age and first investigated the correlation between densitometry and endothelial morphometry in healthy corneas. They found that regardless of age, CD in the posterior layer was inversely and significantly correlated with ECD, CV%, and HEX% [18, 26]. The strong and negative correlation between age and ECD, which has already been proven in the literature, could explain the aforementioned results [26–28]. Similar to their results, Simsek et al. found that the posterior CD in patients with Fuchs uveitis syndrome (FUS), either all concentric annuli or the 0–2 mm zone, was strongly and negatively correlated with ECD (r_s_ = -0.789, *p* < 0.001 in all concentric annuli; r_s_ = -0.545, *p* = 0.005 in the 0–2 mm zone) and HEX% (r_s_ = -0.616, *p* = 0.001 in all concentric annuli; r_s_ = -0.589, *p* = 0.001 in the 0–2 mm zone) [13]. Our results also found that the posterior CD values in the 0–2 mm range were inversely and significantly correlated with ECD (r_s_ = -0.241, *p* = 0.01) and HEX% (r_s_ = -0.157, *p* = 0.031). However, in a retrospective study conducted in China, Zhao et al. found no correlation between CD and ECD in a 4-year observation period after implantable collamer lens (ICL) V4c implantation [29]. There were some differences among these studies. First, the ages were 37.1 ± 6.4, 29.08 ± 5.5, and 61.1 ± 7.6 years in the studies conducted by Simsek et al. and Zhao et al. and in our study, respectively. Second, Simsek et al. enrolled FUS patients with a relatively low ECD (2286.2 ± 283.4 cells/mm^2^); both the study of Zhao et al. and our study enrolled patients without corneal lesions, and the ECD before and after surgery were 2956 ± 235 cells/mm^2^ vs. 2859 ± 211 cells/mm^2^ in Zhao et al., and 2797.3 ± 389.7 cells/mm^2^ vs. 2478.5 ± 257.1 cells/mm^2^ in our study. In summary, we suspected that only when the ECD drops to a certain extent will it cause a statistically significant change in the CD.

Due to a lack of literature on the change in CD after phacovitrectomy, previous studies have shown no significant difference in ECL between phaco- and phacovitrectomy surgery at 3 months postoperatively. Therefore, we referred to cataract surgery-related changes in CD. In our study, the densitometry values were the highest at 1 day postoperatively, then gradually decreased in subsequent follow-up visits but remained slightly higher than baseline at the 3 months follow-up (10.22 ± 0.30 vs. 9.94 ± 0.63), although this difference was not statistically significant. Two studies found the same tendency as that in our study. One was conducted in Taiwan, in which the CD remained slightly higher (*p* > 0.05) than baseline 1 month after phaco-IOL implantation [30]. The other study revealed the same results regarding Fuchs’ endothelial dystrophy (FED) patients at 6 months after cataract surgery [31]. We suspected that this mild increase in CD values might be due to ECL.

Our findings are the first to provide information about corneal parameter changes from the perspective of CD after phacovitrectomy. However, some limitations still exist. The first limitation is the small sample size and the short-term design. Second, this study enrolled only healthy corneas. Hence, we do not know whether this correlation between CD and endothelial parameters applies to patients with corneal pathology. Third, there is a lack of investigation of CD changes after PPV combined with silicon/gas tamponade, which has been proven to be one of the prominent factors. Further prospective studies, including more patients with various corneal conditions undergoing different surgeries are needed to investigate this correlation. However, we still believe that the present study will shed light for future research investigating the effects of intraocular surgery on the cornea and will provide a reference for combined phacovitrectomy or two-step surgery, especially in patients with vulnerable corneas.

## Conclusions

In conclusion, ECD and HEX significantly decreased after phacovitrectomy surgery, and the change in HEX% occurred prior to CV. The CD within all layers in the 0–2 mm zone was positively correlated with CCT. However, posterior CD in the 0–2 mm zone was negatively correlated with ECD and HEX%. CD has the potential to be used as an objective, rapid, and noninvasive tool to reflect corneal health and monitor the process of lesion repair. Understanding the lesion area in advance can effectively prevent iatrogenic injury, especially in patients with subclinical corneal lesions.

## Data Availability

All data used and analyzed in this study are available upon request from the first author: Xin Liu.

## Abbreviations

iFTMH: Idiopathic full-thickness macular hole
PPV: Phacovitrectomy
MH: Macular hole
ECL: Endothelial cell loss
ECD: Endothelial cell density
CD: Corneal densitometry
GSUs: Grayscale units
LOCS III: Lens Opacities Classification System III
BCVA: Best-corrected visual acuity
IOP: Intraocular pressure
OCT: Optical coherence tomography
HEX: Hexagonality
CV: Coefficient of variation
IOL: Intraocular lens
ILM: Internal limiting membrane
CCT: Central corneal thickness
